# Process evaluation of a parent-child communication intervention for adolescent sexual and reproductive health in Uganda

**DOI:** 10.1186/s12889-023-17513-7

**Published:** 2024-01-29

**Authors:** Danielle Fernandes, Elizabeth Kemigisha, Dorcus Achen, Cecilia Akatukwasa, Gad Ndaruhutse Ruzaaza, Gily Coene, Peter Delobelle, Viola N. Nyakato, Kristien Michielsen

**Affiliations:** 1 International Center for Reproductive Health, Department of Public Health and Primary Care, Faculty of Medicine and Health Sciences, Ghent University, Ghent, 9000 Belgium; 2 Faculty of Interdisciplinary Studies, Mbarara University of Science and Technology, P.O. Box 1410, Mbarara, Uganda; 3 Centre of Expertise on Gender, Diversity and Intersectionality, Vrije Universiteit Brussels, Brussels, 1090 Belgium; 4 Department of Community Health, Faculty of Medicine, Mbarara University of Science and Technology, P.O. Box 1410, Mbarara, Uganda; 5 Department of Public Health, Vrije Universiteit Brussels, Brussels, 1090 Belgium; 6 Chronic Disease Initiative for Africa, Department of Medicine, University of Cape Town, Observatory, Cape Town, 7925 South Africa; 7 African Population and Health Research Center, P.O Box 10787-00100, Nairobi, Kenya; 8 Institute for Family and Sexuality Studies, Dept. of Neurosciences, Fac. of Medicine, KU Leuven, Leuven, Belgium

**Keywords:** Sexual and reproductive health, Process evaluation, Implementation science, Parents/caregivers, Young adolescents, Uganda

## Abstract

**Background:**

Previous initiatives concerning adolescent sexual and reproductive health (SRH) education in Low-or-Middle Income Countries (LMICs) have been limited by cultural norms and misinformation perpetuated within families. Responding to the paucity of research on the implementation of SRH interventions in LMICs and limited knowledge regarding their mechanisms, this study undertakes a process evaluation of a parent-focused intervention to promote parent-adolescent communication about SRH in Uganda.

**Methods:**

This paper explores the implementation, contextual factors and mechanisms of impact of the intervention, using the Medical Research Council (MRC) guidelines for process evaluations. Implementation was evaluated through indicators of dose, fidelity and adaptations, acceptability and feasibility. The contextual factors and mechanisms of impact were evaluated to refine the intervention’s causal assumptions. Data was collected during April - October 2021 in South-Western Uganda using a mixed-methods approach, including document analysis, intervention observations, interviews, focus group discussions and most significant change stories.

**Results:**

The acceptability of the intervention was related to its community engagement, the strong rapport with delivery agents, and individual characteristics of participants. Five contextual factors influencing implementation were highlighted; (i) cultural norms, (ii) perceptions about youth SRH, (iii) poverty, (iv) Covid-19 pandemic, and (v) prior research projects in the community. When considering the intervention’s mechanisms of impact, four causal pathways were identified; (i) Awareness of SRH needs helped parents overcome stigma, (ii) Parenting skills training improved SRH communication, (iii) Group learning stimulated shared parenting, and (iv) Group learning improved co-parenting.

**Conclusion:**

The paper presented three key learnings and corresponding recommendations for future research. Firstly, implementation success was credited to meaningful community engagement which improved acceptability and uptake. Secondly, the complex influences of contextual factors highlighted the need for contextual analysis in research studies to inform intervention design. Finally, this evaluation recognised the interplay between mechanisms of impact and suggested further research consider such combined impacts when designing intervention content.

**Supplementary Information:**

The online version contains supplementary material available at 10.1186/s12889-023-17513-7.

## Background

Uganda, a youthful country with about half of the population below the age of 15 years, faces unique challenges in regard to sexual and reproductive health (SRH) [1]. According to the Uganda Bureau of Statistics [1], almost 25% of Ugandan women have given birth before the age of 18 years. Uganda also reports one of the highest rates of HIV prevalence globally, with 5.2% of its adult population living with Human Immunodeficiency Virus (HIV) [2]. Alarmingly, there is a growing proportion of new HIV infections among youth (aged 15–24 years), with girls at higher risk [3]. Global evidence has consistently linked adolescents’ knowledge on SRH to health outcomes, with lack of accurate information being recognised as one of the prominent contributors to engaging in risky sexual behaviour [4]. Uganda’s Demographic and Health Survey found that only 40% of adolescents (aged 15–19 years) have sufficient knowledge related to HIV prevention. Low levels of knowledge about menstruation, reproductive hygiene and condom use have also been reported [1, 5].

Comprehensive sexuality education (CSE) has been highlighted to address this information gap among young people by equiping them to make informed decisions regarding their sexual and reproductive health and rights [6]. In Uganda, previous CSE efforts faced challenges due to societal resistance to topics like adolescent sexuality, homosexuality, gender identity, reproductive justice, abortion, positive sexuality, and masturbation, all integral parts of the CSE curriculum [7, 8]. Such resistance has both religious and cultural facets, with religious leaders framing CSE as an attack on moral values, while political leaders invoke Uganda’s colonial past to describe CSE as ‘cultural imperialism’ [9, 10]. This resistance has influenced political approaches and policy implementation, with a temporary ban on non-abstinence sex education in 2016 driven by parental concerns about corrupting their children [11]. Although the ban was lifted in 2018, the cultural context continues to limit the effective implementation of sex education policies [8, 12].

Mbarara University of Science and Technology (MUST, Uganda) and Ghent University (UGent, Belgium) collaborated on an evidence-based CSE program in Ugandan schools [13]. However, an evaluation revealed limitations in the school-based intervention, attributed to gender stereotypes and misinformation within families [13, 14]. Recognizing the importance of involving families, a participatory parent-focused communication intervention was initiated to promote sexual and reproductive health (SRH) in rural Uganda. While initial findings from the outcome evaluation suggest success (with results currently in the process of being published), there remains a gap in understanding the mechanisms and reasons behind the effectiveness of such interventions.

There is a growing awareness of the influence of context on a program’s success, with the contextual ‘fit’ of an intervention being instrumental to its acceptability, uptake and achieving desired outcomes [15]. Nevertheless, there is a reported lack of process evaluations covering the implementation of health care interventions in low and middle-income countries (LMICs) [16, 17] which speaks to the long tradition of transplanting successful approaches from high-income countries (HICs) without regard to contextual influences [18]. This scarcity of process evaluations is also observed when it comes to sexuality education interventions in LMICs [19]. This is particularly relevant in contexts like Uganda where religious and social norms stigmatising premarital sex present a challenge [20]. In such cases, it is important to understand the factors contributing to acceptability and successful implementation of the intervention. Generating a knowledge base for implementation processes in the real-world is an important step to successful scale-up and can act as a road map for implementers in similar contexts.

Process evaluations provide valuable insight into the contextual factors and causal pathways that determine a program’s success, allowing us to open the “black box” of intervention effectiveness [21]. This is particularly important for complex interventions which consist of different components and dynamic interactions between individuals involved in the intervention [21]. The outcomes of such complex interventions may be determined by various contextual factors and by the implementation process itself. By integrating a process evaluation alongside the intervention delivery, it is possible to draw out the causal mechanisms of the intervention, identify the active ingredients and interpret the intervention outcomes.

This study aims to address (i) the paucity of process and implementation research on SRH interventions in LMICs and (ii) the limited knowledge regarding the mechanisms of impact of such interventions, through a process evaluation of the parent-child SRH communication intervention in rural Uganda. By providing an understanding of how this complex intervention works, the findings from this study can be used to successfully reproduce SRH interventions in similar contexts. The objectives of the present study are hence:

(i) To assess the implementation process of a parent-child communication intervention to improve adolescent SRH in the rural Ugandan context; and,
(ii) To study the contextual factors and mechanisms of impact of the intervention.

## Methods

**Fig. 1:**
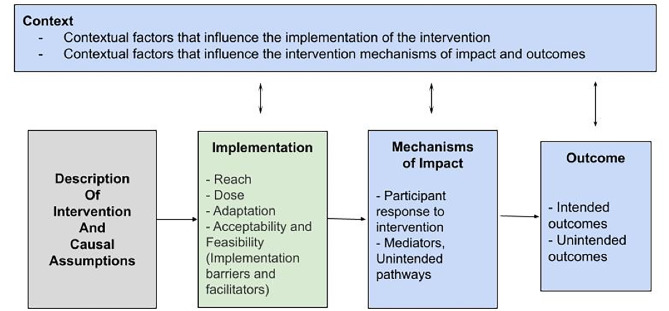
Process Evaluation Framework adapted from MRC guidelines [17]

The process evaluation was based on a mixed-methods approach to assess indicators of the intervention implementation and causal assumptions. The UK Medical Research Council (MRC) framework for the process evaluation of complex interventions [22] was used to guide the study methodology. The adapted evaluation framework is depicted in Fig. 1.

The first study objective of assessing the implementation process, was addressed through understanding what was delivered and how this was delivered. Implementation was studied by assessing reach (the population who accessed the intervention), dose (the quantity of the intervention that was delivered), adaptations (changes made to the intervention design), acceptability and feasibility of the intervention. Indicators of dose and reach were measured quantitatively, while adaptations, acceptability and feasibility were assessed qualitatively (see 2.3).

The second study objective was addressed by studying the contextual factors and mechanisms of impact which influenced the intervention outcomes. Context refers to external factors that influence implementation, the intervention and potential outcomes. Mechanisms of impact focus on participants’ responses to the intervention and other mediators which influence outcomes. Contextual factors and mechanisms of impact were explored qualitatively.

### The intervention and its causal assumptions

The intervention was delivered by the Mbarara University of Science and Technology (MUST) research team in Mbarara, Uganda over a period of 6 months. Mbarara is a south-western municipality of Uganda, with a population size of approximately half a million residents, of which almost 50% are below 18 years of age [23]. The study was carried out in six villages; Muko, Rwebishekye 1, Rwebishekye 2, Kaburaishokye, Kikoma, and Mishenyi, which are close-knit rural communities with limited SRH services [18].

Prior to the commencement of the study, the research team conducted extensive networking within the study sites through setting up a Community Advisory Board to guide the project and involving community members in the development of the intervention. Several community members, particularly those who are parents of children aged 10–14 years, were included in the intervention development phase of the project. Community members were also involved in the delivery of the intervention. The delivery agents of the intervention (called community facilitators) consisted of prominent community members, including religious leaders. One facilitator was appointed from each community and was trained to deliver the intervention sessions to other parents in the community. Additional community members were appointed as community mobilisers, who informed the community about the intervention and encouraged parents to participate.

The intervention was delivered over a 6 month period and consisted of two phases (Table 1): 3 modules focusing on positive parenting, cultural values, and adolescent SRH; and five modules focusing on communication, puberty, relationships, sexually transmitted infections (STIs) and pregnancy prevention. The intervention sessions were highly interactive with group discussions and role-plays aimed at encouraging participants to reflect on their values and practice new skills.

**Table 1:** Overview of intervention content

Intervention Module	Description	Outline
Training of Community Facilitators	Community facilitators trained on verbal and non-verbal communication skills, moderation of culturally sensitive discussions, and provision of judgment-free CSE.	Training of trainers- 15 sessions
PHASE 1- PARENTING ADOLESCENTS		
Module 1: Positive Parenting	Builds parenting skills by encouraging relationship-building, communication and the use of positive reinforcement.	3 sessions
Module 2: Cultural and Religious Values	Enables reflection on cultural, religious and social norms regarding parenting, and critically evaluating the influence of these norms.	2 sessions
Module 3: Adolescence and SRH	Informs parents about the process of adolescence, including physical development, onset of puberty, societal, emotional and health challenges and sexuality.	2 sessions
PHASE 2- PARENT-ADOLESCENT COMMUNICATION ON SRH		
Module 4: Communication skills	Equips parents to create a supportive environment for effective communication, using role-plays to prepare for discussions on sensitive topics.	2 sessions
Module 5: Puberty	Provides information about puberty and related challenges, and enables parents to support children to transit puberty in a healthy manner.	2 sessions
Module 6: Relationships	Informs parents about healthy and unhealthy relationship patterns, and equips them to communicate with children about relationships.	1 session
Module 7: Sexually Transmitted Infections (STIs)	Provides information about HIV and other STIs, including adolescent vulnerability, disease symptoms, and preventive and self-care practices. Equips parents to effectively communicate about STI prevention.	1 session
Module 8: Pregnancy Prevention	Informs parents of the challenges of teenage pregnancy and and equips them to effectively communicate about pregnancy prevention.	1 session
COMMUNITY ‘EXPERT’ TALKS		
Community-based talks on safety and prevention of selected adolescent health risks	Community talks were organised, with experts invited to deliver information on specific SRH topics: • Sexuality and sexual behaviour • Sexual violence, recognition and reporting • Gender, sexuality and human rights • Emotional and mental health- building self-esteem, and addressing depression and addictions • Entrepreneurship skills for parents	5 sessions

The main objective of the intervention was to improve parent-child communication around SRH. The intervention was assumed to achieve improved parent-child communication on SRH through:

(i) Increasing parents’ awareness of adolescents’ SRH needs.
(ii) Improving parents’ communication skills around SRH, and.
(iii) Facilitating communities to support parental engagement in SRH communication.

**Fig. 2:**
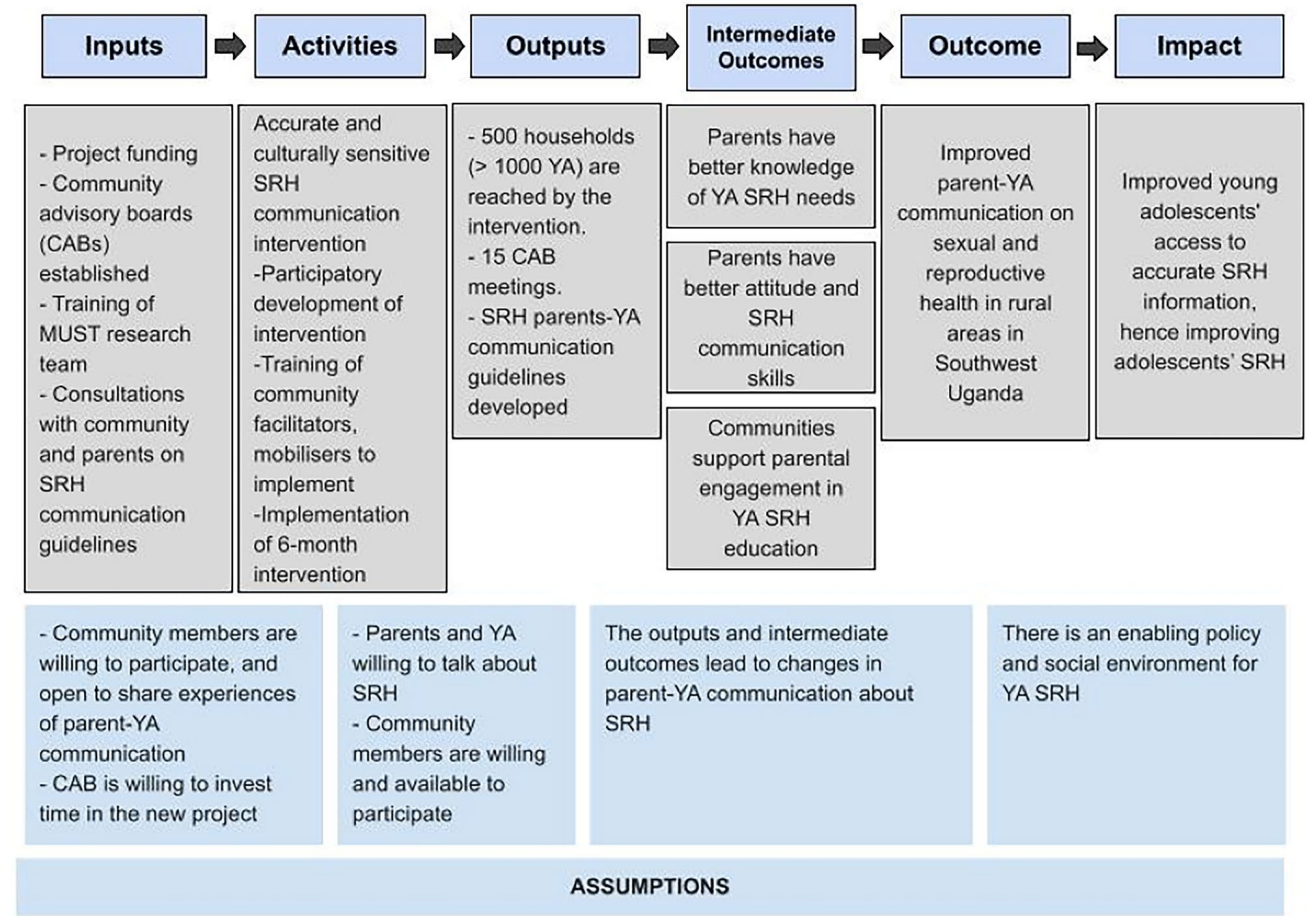
Initial logic model of intervention implementation and its causal assumptions

The logic model developed in the formative stage of the study regarding the impact of the intervention and the implementation of the intervention was used as a reference point (depicted in Fig. 2). The causal assumptions of this initial model were tested and refined through the process evaluation.

### Intervention participants

The criteria for participating in the intervention was (i) a primary caregiver (biological parent, stepparent, aunt, uncle, or grandparent) of children aged 10–14 years, and (ii) resident of one of the study sites (Muko, Rwebishekye 1, Rwebishekye 2, Kaburaishokye, Kikoma, and Mishenyi). Participants were recruited through networking within the community. The research team reached out to community members who had participated in the formative phase of the study and invited them to receive the intervention. Initially, 174 community members were recruited through this approach. Additionally, the intervention facilitators and mobilizers disseminated information about the intervention and encouraged an additional 113 community members to join the study. In total, 287 participants were enrolled to the study and received the intervention.

### Ethics

The study received ethics approval from the Institutional Research Ethics Committee of Mbarara University of Science and Technology (reference number 15/05–19) and the Uganda National Council of Science and Technology (reference number SS 5108). All intervention participants, researchers and delivery agents provided informed consent to participate in the study and agreed to have their interviews audio recorded and excerpts used for publication. Participant anonymity was maintained when reporting data from the study by removing identifying information. Data logs with identifying details of participants were stored securely and only accessed by the research team.

### Data collection and analysis

**Fig. 3:**
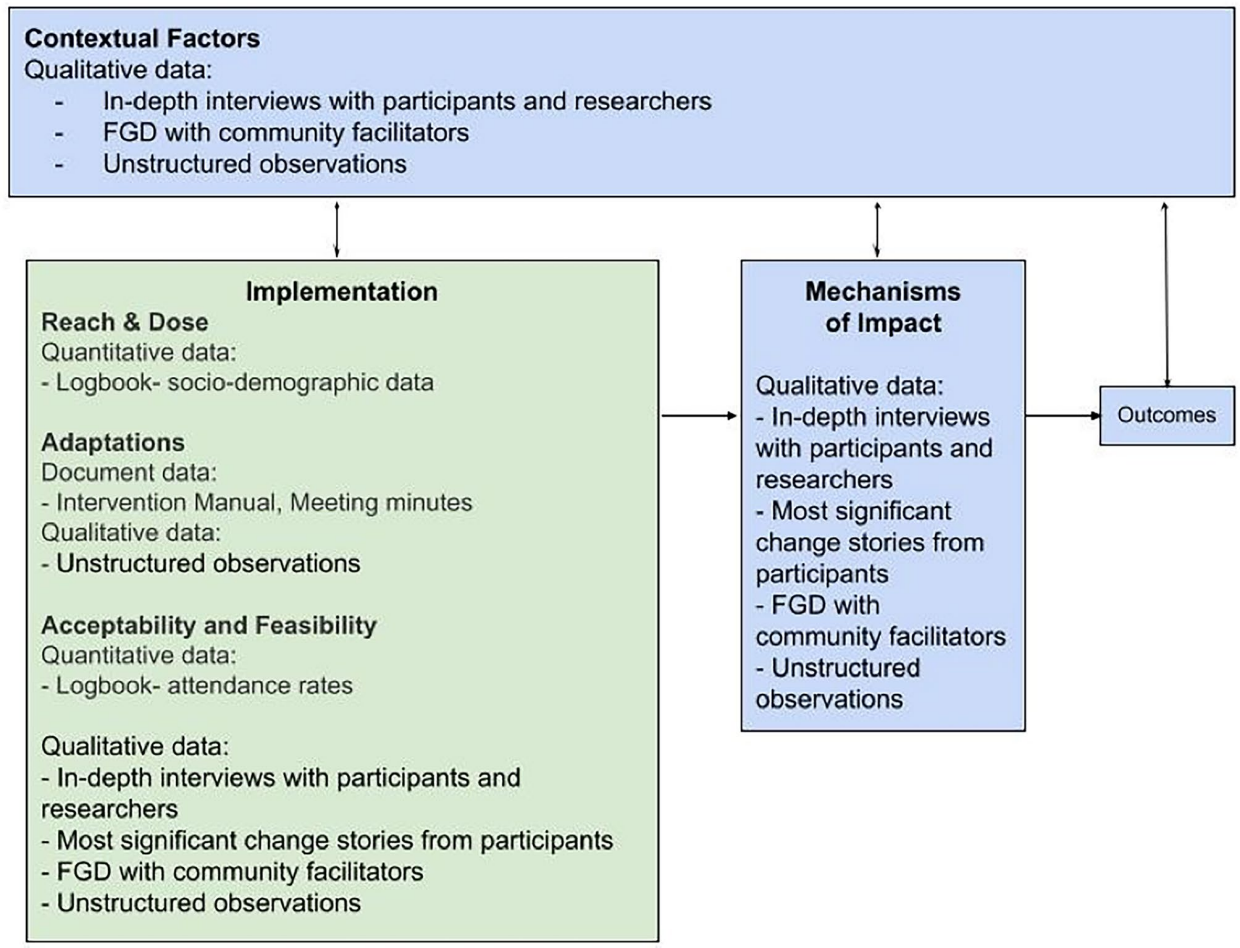
Overview of data tools and sources corresponding to process evaluation indicators

Data collection was conducted during April - October 2021, and comprised of four interviews with intervention participants, three interviews with researchers, one focus group discussion with community facilitators, 30 stories of most significant change from intervention participants, 24 observations of intervention sessions, a logbook of participant attendance and the minutes of the implementation team’s meetings. Data was collected from multiple sources to measure the indicators outlined in the evaluation framework. Figure 3 presents an overview of the data sources and data tools related to each process evaluation indicator.

(i) Quantitative data: A logbook was maintained to record participants’ sociodemographic details and attendance at each session. An overview of sociodemographic details of the participants is presented in Table 2 and attendance rates are presented in Table 3.
(ii) Document data: The intervention manual, notes from the intervention facilitators and minutes from the project meetings were documented and compiled. These documents were reviewed to identify adaptations made to the intervention and the reasons for these adaptations (presented in Table 4).
(iii) Qualitative data: Data was collected through unstructured observations, in-depth interviews (IDIs), Most Significant Change (MSC) stories, and focus group discussions (FGDs).
  A. Unstructured observation- Unstructured observations of 24 intervention sessions were conducted to explore implementation, mechanisms of impact and contextual factors. Data from the observation notes was extracted into summary tables describing acceptability and feasibility, adaptations to the planned delivery and content, and contextual factors influencing the intervention.
  B. In-depth interviews- Interviews were conducted with intervention participants and researchers implementing the project. The interviews lasted approximately 45–60 min, and the interview guides can be found in the appendix (Appendix A and B).

- Interviews with intervention participants- Four In Depth Interviews (IDIs) were conducted with a sample of intervention participants to discuss their experience of the intervention, contextual factors influencing implementation and outcomes, and mechanism of change. Intervention participants were purposively sampled for inclusion in the process evaluation. In order to include diverse perspectives in the evaluation, participants of different genders, from different villages and with different levels of engagement (from regular attendance to drop-outs) were identified.
- Interviews with researchers- Three IDIs were conducted with community facilitators and research team members to discuss their views on adaptations made to the intervention, acceptability, feasibility and contextual factors influencing the intervention delivery.
  C. Most Significant Change (MSC) stories- The MSC technique is a participatory method of collecting stories of change experienced by participants through individual interviews, which is then subjected to selection by stakeholder groups [24]. Participants are asked to describe an outcome that represented their most significant change and explain the personal significance of it. The MSC guide used to structure these interviews can be found in the appendix (Appendix C). 30 MSC interviews were conducted and the interviewers used neutral framing of the questions to minimise bias due to social desirability. In this evaluation, 30 stories of change obtained from intervention participants at the end of the intervention were used to explore mechanisms of impact related to outcomes experienced.
  D. Focus group discussions (FGDs)- A FGD was conducted with six community facilitators to discuss their views on adaptations made to the intervention, acceptability and feasibility of the intervention, contextual factors influencing implementation and outcomes, and mechanisms of impact. The FGD was approximately 90 min long, and the guide can be found in the appendix (Appendix D).

Data from IDIs, FGDs and MSC stories were recorded and transcribed verbatim. Interviews in local languages were transcribed and translated to English. The transcribed interviews, MSC stories and observation notes were analysed using NVivo version 11. A mixed inductive and deductive thematic analysis approach [25] was used, whereby the evaluation framework guided the coding process while also allowing for new themes to be generated from the raw data. Two independent coders (DF and AD) first familiarised themselves with the data by reading a sub-set of the interviews and generating preliminary codes. Through a discussion between the two coders, these initial codes were reviewed in line with the evaluation framework. This discussion fed into the development of an initial codebook, which was used to code the remaining data. The coding process was reflexive and iterative, with points of reflection between the coders and the rest of the evaluation team leading to refining the codebook. A full overview of the codes and themes from the qualitative interviews are presented in the appendix (Appendix E).

## Results

This process evaluation studies the implementation of the intervention by providing an overview of the dose, reach, adaptations, and its acceptability and feasibility (Sect. 3.1). The causal assumptions for the intervention are also explored and refined, by outlining the mechanisms of impact and identifying contextual factors that influence these, as well as describing unexpected causal pathways.

### Implementation

#### Dose and reach

The intervention was delivered in six rural communities to a total of 287 participants. The socio-demographic characteristics of the participants are presented in Table 2.

**Table 2:** Overview of participant characteristics

Participant characteristics		Participants (n = 287)
Gender	Female	216 (75.5%)
	Male	71 (24.8%)
Age * (median; age range: minimum - maximum)		44; 17–83 years
Village**	Kaburaishokye	62 (21.6%)
	Rwebishekye	39 (13.6%)
	Mishenyi	103 (36%)
	Muko	41 (14.3%)
	Kikooma	42 (14.6%)

*There is missing age data for 42 of the 287 participants, due to challenges in data collection. A wide age range was seen among the participants, with young adult and adolescent participants attending the intervention as caregivers. The younger participants were not necessarily biological parents, but represented caregivers (such as aunts, step-parents)

** The intervention was originally planned to be delivered in six rural communities, however data from Rwebishekye 1 and Rwebishekye 2 were merged and reported together

The intervention consisted of two phases. The first phase was delivered over four months, and consisted of four modules. Each module was delivered over one to three sessions. The second phase, delivered over one month, consisted of four modules. Each of these four modules was delivered through a single session. The intended duration of each session was approximately 60 min. However, due to occasional delays in commencing the sessions, the delivered intervention sessions had a variable duration of 40–60 min. The first phase recorded higher attendance rates, with a drop in participation during the second phase. This coincided with the increase of Covid-19 cases in Uganda at this time and the intensification of lockdown measures. There was higher female participation throughout the intervention, which was consistent with gendered parenting norms in the context. Additionally, male caregivers had limited time to participate in the intervention due to work commitments. Table 3 presents an overview of participant attendance for each intervention session.

**Table 3:** Overview of participant attendance for each session

Intervention Session				Participant attendance * n (%)	Female participant attendace ** n (%)
Phase 1	Module 1	Parenting	A	146 (50.8%)	116 (79.4%)
			B	160 (55.7%)	130 (81.2%)
			C	135 (47%)	103 (76.2%)
	Module 2	Socio- Cultural norms	A	139 (48.4%)	109 (78.4%)
			B	111 (38.6%)	90 (81%)
	Module 3	Adolescence	A	121 (42%)	95 (78.5%)
			B	133 (46.3%)	106 (79.6%)
	Module 4	Communication		110 (38.3%)	81 (73.6%)
Phase 2	Module 5	Puberty		98 (34%)	76 (77.5%)
	Module 6	Relationships		106 (37%)	79 (74.5%)
	Module 7	HIV/ STIs		102 (35.5%)	77 (75.4%)
	Module 8	Pregnancy prevention		118 (41%)	89 (75.4%)
Average attendance rate (over the intervention)				42.9%	77.6%

*The participant attendance rate describes the percentage of participants attending each session of the total 287 participants enrolled in the study

**The female participant attendance rate describes the percentage of female participants attending each session of the total participants attending that session

#### Adaptations

Data from the observation notes, meeting minutes and interviews reported some deviations from the planned delivery of the intervention. Intervention sessions occasionally started later than planned due to bad weather conditions and transportation issues. This impacted the delivery of the intervention, as delivery agents attempted to cover the session in a shorter period. The Covid-19 pandemic also led to major changes in the intervention structure, with the second phase of the intervention being delayed in response to the sudden lockdown. Intervention content was also shortened and delivered over fewer sessions, and adapted to reduce close interactions due to Covid restrictions. Adaptations made to the intervention are described in Table 4.

**Table 4:** Adaptations to intervention delivery and content

Adaptation made to the Intervention		Reason for adaptation
Intervention Delivery	Phase 2 was originally planned to be delivered from July onwards. There was a delay and Phase 2 was delivered in September.	Uganda went into lockdown in June as a restrictive measure against the Covid-19 pandemic. This delayed the commencement of Phase 2.
	Phase 2 was originally intended to be delivered over 3 months, with each module consisting of 2–3 sessions. However, Phase 2 was shortened to be delivered over a single month. The 4 modules were shortened and delivered over a single session each.	The modules in Phase 2 were shortened to limit contact time within the community, in light of the rising Covid-19 cases in the country. Covid restrictions in place could only allow for fewer numbers per group and shortened contact time for delivery of the intervention
	There were often delays in starting the sessions. This left the facilitators with a shorter amount of time to complete the activities and learning planned for the session.	Delays in starting the session were due to unfavourable weather conditions which led to community members or the intervention team arriving late.
Intervention Content	It was originally planned to deliver additional sessions on SRH topics to complement the intervention. These additional sessions were expanded as ‘expert talks’, where experts were invited to the community to deliver lectures on a range of topics. Five talks were arranged on (i) adolescent sexuality, (ii) sexual and gender based violence, (iii) gender and human rights, (iv) mental health and (v) entrepreneurship skills.	During the intervention, participants discussed topics related to adolescent’s health and recognised that they have limited knowledge about these issues. They also pointed out wider issues in their community, such as poverty, unemployment and violence, They requested additional information to be delivered for the whole community about these issues.
	An adaptation was made to the ‘Value Clarification’ activity in the parenting module. The activity required parents to reflect on the values they hold regarding sexual health topic (eg- teenage pregnancy, abortion). The participants were asked to write a description about the values they hold. However, the format was changed to allow for an open discussion about values.	The participants were initially asked to write their reflection, due to concerns that participants may be hesitant to voice controversial opinions in a group setting. However, this was considered inconvenient as some participants could not write or had trouble expressing themselves through writing. The participants preferred to have an open discussion. They engaged in a lively discussion and were able to speak freely.

#### Acceptability and feasibility

The qualitative data showed that intervention participants provided positive feedback regarding the intervention, indicating its acceptability in the study setting. Participants expressed appreciation and gratitude that the study intended to improve adolescent health in their community and believed it met their needs. Participants also demonstrated active engagement during the sessions with lively discussions taking place.

“The program is useful because it bridges the gap between the parent and the child… it connects them. That is the only way that free conversation can start to follow and when you understand your child, it becomes easy to nurture him or her into a better person in future.” - Participant (Female, 35 years).

When reflecting on the program acceptability, interview participants described three factors as key to its feasibility and acceptability: the engagement with the community during intervention development and implementation, the rapport with delivery agents, and personal factors of the intervention participants.

#### Community engagement

The participatory approach of the study which involved engagement with the community to co-create the intervention was considered instrumental in improving its acceptability and feasibility. The time, duration and location of intervention sessions was scheduled to align with the availability of community members. Tailoring of intervention activities through contingency plans for illiterate participants, using contextually appropriate language and aligning the intervention with religious and cultural values, contributed to acceptability in the community.

“So, the community based participatory approach allowed us to bring these religious leaders on board. So having all these kinds of people on the project has given us a go ahead smoother than any of us expected.” - Researcher.

“I remember when we were in the community, the members of the community advisory board would tell us to adjust to certain things like language because even the word sexuality itself is not direct, you might find it has like three words in Runyankole, the language of the land. ” - Researcher.

Prior to commencing the study, sensitisation sessions were organised to provide context regarding the objectives of the intervention. This helped to dispel negative reactions to the focus on adolescent sexuality which was stigmatized in this setting (rural Uganda). It also functioned as a method to clarify what participants would receive from the study and helped to reduce expectations for material or financial support.

“In the start, there were so many questions especially to the community leaders, because, at first, people were so used to their traditional beliefs and laws. So when they heard that the project is being brought by a group from the university and some foreigners, they were scared that their children were going to be taught the foreign culture. But after being sensitized, their negative attitude towards the project changed.” - Community Facilitator.

The study was embedded in the community, with community members being recruited to deliver the intervention sessions (as community facilitators) and to spread awareness about the study (as community mobilisers). This was considered a facilitating factor which motivated participants to attend. The community facilitators represented different backgrounds, including religious leaders, which helped to broker acceptability.

“We had a pastor, for example, who was very bold about these SRH issues. He would boldly talk about sexual and reproductive health. People thought, ‘Ohh, maybe it’s not religiously acceptable to talk about these things’, but he made people in the community realize that this is the reality that is taking place in our community, to know that they [adolescent pregnancy, HIV and other SRH issues] are there, we have to accept that they are there and deal with them.” - Researcher.

#### Rapport with the delivery agents

The participants reported feeling comfortable with the intervention delivery agents due to their friendly approach which engaged participants as active contributors in the sessions. Being members of the community, they had a better understanding of the context and were accepted easily by the participants.

“The first time I attended, I saw exactly what was being taught which was so good and also the facilitators themselves were so passionate. They like us and are so nice. They made us feel at peace. They explained each and everything very well, they spoke in the language that we understand very well, gave us time to ask and share knowledge among ourselves. The facilitators were generally so friendly and close to us.” - Participant (Female, 57 years).

#### Personal factors

Participants described their work schedules or life circumstances as facilitating factors to attend sessions. Several participants also cited their personal motivation as a supporting factor. This was particularly the case among older participants who viewed the intervention as a chance to educate themselves and took pride in this.

“Personally, I grew up with a spirit of wanting to be educated but I never got the chance because my parents couldn’t afford to take me to school, so when I heard about this program and chance, I was very interested and now I am having the opportunity of learning.”- Participant (Male, 61 years).

### Context

Interview participants highlighted five focal contextual factors that impacted the implementation of the intervention. Three key themes emerged, centred around pre-existing factors; (i) cultural norms around parenting, (ii) perceptions about youth SRH, and (iii) the interplay between poverty and parenting. As the intervention was delivered during the Covid-19 pandemic, the latter also emerged as an important contextual factor (theme iv). Finally, the legacy of other research studies that were conducted in the communities was highlighted as a factor that influenced implementation (theme v).

#### Parenting norms

The social context of the communities had an influence on the intervention implementation and participant responses. Stereotypical gender roles around parenting impacted the participation of male caregivers, with parenting often considered the woman’s responsibility. This gender imbalance also manifested in women needing permission from male partners to attend the sessions, which negatively impacted participation.

“Men are still a challenge in our community, they do not care and they do not have time for their children. Even when you come from attending the [intervention] session and you explain to him whatever you are taught, you do not see them bothered at all. They do not have time for such and they abandoned the role of parenting to us the mothers.” - Participant (Female, 35 years).

“Some of the women are going to be prevented by their partners because we are in a patriarchal society, women have to get permission from their partners to come and attend. Because I saw some incidents were some women were not coming because their husbands had prevented them from coming.” - Researcher.

Parenting approaches were also embedded in the cultural context, with authoritarian parenting considered the norm. While the intervention was largely successful in changing the mind-set around parenting, these long-held parenting norms were still evident in the narratives. Participants often described keeping tabs on their children’s movements, setting curfews or restrictions, and disciplining neighbourhood children.

“In short, I never knew anything about the stories of my children. I was a rigid and principled parent and too tough. Actually, my children used to fear me.” - Participant (Female).

“Personally I have already started implementing and applying what I learn from the program. For example, if I come across a girl child or a more roistering around or messing around in our community, I do not keep a blind eye and let them. But instead I have to intervene, advise or discipline them accordingly.” - Participant (Male, 61 years).

#### Adolescent SRH perceptions

As adolescent sexuality was highly stigmatised, open discussions about sexual health were limited with many participants describing “fear” to discuss the topic with their children. This climate of fear around sexuality influenced the study outcomes, with some participants expressing that their children were hesitant to engage in a conversation about SRH.

“When we were growing up, it was never heard off and I had never spoken with my mother about issues to do with sexual reproductive health. We used to fear such issues so much, in the end the children would get challenges.” - Participant (Male, 38 years).

Before the intervention I used to fear talking to my children about Sexual and Reproductive health, or adolescence. My children would fear talk to me too, because I wasn’t close to them.

- Participant (Female).

Furthermore, the negative attitudes towards sexuality influenced the way participants discussed SRH with their children, with many using a risk-focused perspective and emphasising the potential dangers of sexual activity.

“I told them that there are many bad things that originate from practicing sexual intercourse, have you heard about them and they answered that they know and have heard that on the radio that you can get diseases and suffer from HIV/AIDS.” - Participant (Male).

#### Poverty

The backdrop of poverty, food insecurity and limited access to resources influenced the intervention. Participants often had limited time to spend with their children and expressed concern that poverty would influence their children to make bad decisions.

“When we were taught, I realized that we haven’t been responsible parents to our children. we didn’t have the time and responsibility to know where the children would be going.” – Participant (Female).

“…a girl will start taking money from men so as to have what her other friends have, but if I have money and I am able to support her, then she will not envy other people’s things.” – Participant (Male, 61 years).

#### Covid-19 pandemic

The intervention was delivered during the Covid-19 pandemic, which negatively impacted attendance rates due to fears of contracting the virus and government restrictions on movement. The lockdown also had economic consequences of food and income insecurity which reduced participation.

“It [Covid-19] really affected us seriously. movements are so restricted and because of that our facilitators from Mbarara University find it difficult to get into our communities. In addition to that we are not allowed to gather in groups because of social distancing as a way of ensuring COVID SOPs… Whenever it rained during the training sessions, we would overcrowd in a room and it would seem uncomfortable… remember Covid-19 was still with us. Others used to cough.” - Participant (Female, 57 years).

The media coverage around adolescent pregnancies and sexual violence being exacerbated during the pandemic had the unexpected effect of heightening awareness of SRH challenges in the community. This was considered an influencing factor in boosting community members’ motivation to participate in the intervention.

“Because of the lockdown and children not going to school, there were a lot of negative SRH outcomes, girls got pregnant, they dropped out of school, there was a lot of sexual violence in the community and many other negative SRH outcomes in the community. And these came as a result of the lockdown and the COVID-19 outbreak. So, as a result of this, I think this also maybe in a way probably could have influenced a change in mindset of the people in the community and the need for SRH communication with children.” - Researcher.

#### Communities as intervention settings

The study sites having previously hosted other research studies, influenced how the community members viewed research activities. Some had seen prior interventions fail, making them skeptical of the present study. Prior research activities were also accompanied by liberal cash incentives which created expectations for financial support or material ‘gifts’ among the community.

“We have been introduced to many projects in our area, some projects have continued and others have failed because probably in the course of the project, things are ruined. So, the fact that people knew about most projects coming and failing, they thought that this project was also going to fail but up to now the project is still going on.” - Community Facilitator.

“When the community sees us, they get high expectations. I remember one of the parents said, “I expect to get a bursary or tuition for my child”, so they expect so much as if we are the government or an angel that has come to give our money or something.” - Researcher.

### Mechanisms of impact

When considering the mechanisms of impact of the intervention, the three causal assumptions outlined at the onset of the study were examined and further refined. An additional pathway was identified which described how intervention learnings facilitated parenting to be viewed as a combined effort of both male and female caregivers.

#### Overcoming stigma through awareness

The implementation team described strategically providing information on the SRH challenges faced by youth to challenge societal norms and stigma around SRH discussions. This was reflected in participant narratives, which described the intervention content as a turning point where they recognised the pressing need to address SRH issues with their children despite the societal stigma around these conversations. By triggering awareness to the potential threats to their child’s health as well as the child’s unmet need for information, the intervention motivated parents to initiate SRH discussions. This was coupled with accurate and comprehensive SRH information content, thus allowing parents to initiate well-informed conversations with their child.

“For example, one of the sessions was to display problems that are going on in our communities as far as Sexual and Reproductive health is concerned. In the other session they were having a debate of how can a parent give her child contraceptives, saying it’s like you are telling them to go and have sex. But now, in this session we are making them face the reality that actually girls are getting pregnant, so what are you going to do to prevent these pregnancies.” - Researcher.

“But for the good of the parents towards good parenting there was no part that was not necessary or difficult for us to take in. It is true, it was not common to talk about such topics about sex in our community but after we got the training and how important it is to talk to our children, there is nothing we are afraid of anymore.” -Participant (Female, 35 years).

#### Re-thinking parenting as friendship

The implementation team reported that the parenting module encouraged parents to evaluate their priorities as parents and recognise that children need holistic care that goes beyond providing basic needs. The intervention facilitated reflection on parenting styles and its impact on engaging with the child. Participants reported that this activity triggered a realisation that their authoritarian parenting style hampered communication and relationships with their children. Several participants described treating their children as friends, with some introducing acts of gratitude into daily interactions, making time to spend talking, and replacing punishments with positive reinforcements.

“The things that we study that brought change in my life is one we started with studying that a child is like a plant and for a plant to grow well there a certain things one need to do therefore we learnt that you should care for the children and give the time to understand them better and this helped me because I used this technique on my child.” - Participant (Female).

“An authoritarian parent is not helpful because the more authoritarian the parent becomes, the child will not be ready to share what they are going through because the parent is so tough and so strict all the time. So they differentiated role-model parenting with authoritarian parenting. Role model parenting is a person who understands a child, takes in the view of a child and guides the child. So I think with that, they came to realize that, “maybe if I am becoming very strict with my child, then my child will not open up”. So I think they have picked new values.” - Researcher.

#### Shared learnings and shared responsibilities

The group setting, open discussions, interactive activities and participatory approach used during the intervention sessions facilitated sharing of learnings and ideas between participants. Finding a common ground throughout the intervention also motivated participants to support each other as parents, with parenting being recognised as a communal responsibility.

“First of all the program mobilises a lot of people and brings them together, they teach you as a group, give you an opportunity to share experiences, advise one another and find better solutions to address the challenges of how best to parent a child without fear.” -Participant (Male, 38 years).

“We came to understand and appreciate our culture and our traditional ways of raising our children. That we have to take a collective responsibility and the children in our community, to know and understand all the children and be able to advise and discipline them were necessary so that we can get better children of tomorrow.” -Participant (Male, 61 years).

#### Unintended pathway: gendered parenting to co-parenting

Participants described changes in co-parenting approaches, with the intervention encouraging parents to work together as a team. This changed the narrative around gendered parenting roles and supported fathers to take a more active role. Participants also reported changes in their personal lives, with improved communication and relationships with their partners.

“The program taught us that when both parents, that is father and mother, team up, they can easily raise their children very well. For example, for their education, both parents contribute towards their school fees and buying them clothes. Therefore when it comes to advising and parenting, one parent cannot do it alone properly but when it’s done together as father and mother, you cannot go wrong.” -Participant (Female, 57 years).

“The biggest thing I learned is how to handle my family because I never saw any reason of having a discussion with my wife on how I spend the money I earn or plan with her what to use this money for. As long as she told our child’s school fees is 300,000 and I pay it that’s it. But now, after the study, I would even return home and discuss for her whatever we learned.” - Participant (Male).

## Discussion

This study was a mixed-methods process evaluation of a parent-child communication intervention for sexual and reproductive health in rural Uganda. The study had the dual objectives of exploring the implementation process of the intervention and outlining the contextual factors and mechanisms of impact that influenced its success. Three key learnings emerged from this evaluation which are presented below with corresponding recommendations for future research.

*Implementation success through community engagement and flexibility*- The implementation team heavily invested in community involvement; by adopting a participatory approach which allowed community members to contribute to intervention content, through recruitment of community members to spread awareness and deliver the intervention, and by frequent consultations with community representatives during the program. This investment paid off with communities showing high acceptance of the program, which helped to navigate challenges posed by the pandemic and the cultural stigma around sexuality. The importance of whole-of-community engagement as a method to overcome stigma and promote uptake of SRH interventions is echoed in the global literature [26], with participatory approaches often hailed as the pathway to meaningful engagement [27]. The study encountered minor disruptions due to bad weather and unreliable transportation which typically occur in the local context, as well as unexpected set-backs due to the COVID-19 pandemic. These were overcome through flexibility by the implementation team. Furthermore, the goodwill developed within the community also ensured their willingness to accept program adaptations. Global implementation science has described the utility of community engagement in overcoming challenges encountered during program implementation [28]. This indicated the importance of brokering meaningful community engagement as well as embracing design flexibility to ensure implementation success. Thus, it is recommended that future interventions invest in whole-of-community engagement and leave space for flexibility in intervention delivery. Such community ownership through community-based participatory approaches have positive implications for long-term intervention success and sustainability.

*Context works in mysterious ways-* With Uganda being a patriarchal, deeply religious society, it was unsurprising that communities held conservative views on adolescent sexuality and favoured authoritarian parenting practices. These influences continued to manifest during the intervention and in participant narratives of positive outcomes, with reports of successfully overcoming stigma to initiate SRH discussions still bearing a risk-focused perspective. Prior studies in Sub-Saharan Africa have described how stigma and fear dominate parent-child SRH discussions, thus hindering open discussions [29, 30]. Evaluations on health interventions have reported how long-held socio-cultural beliefs persist and influence intervention outcomes [31, 32], which speaks to the need to address these factors when designing intervention content. Interestingly, some contextual factors had dual and contradictory influences on implementation. For example, while the pandemic dissuaded participation, exacerbation of SRH challenges during this period boosted awareness and motivated attendance. Similarly, while prior studies in the communities helped researchers to broker acceptability through existing networks, they also came up against financial expectations and skepticism created by previous projects. This complex influence of contextual factors has been reported in prior health intervention studies [33, 34], with one study on SRH interventions in Ethiopia calling for SRH interventions to broaden their ambit to address these influences [29]. Thus, rather than looking at context as either positive or negative influences, it is important to understand that contextual factors interact with interventions in complex ways to impact implementation and outcomes. While the importance of context has previously been emphasised when developing complex interventions [35, 36], this study further recommends in-depth explorations of the persistent, manifold and potentially paradoxical influences of the context when developing a health intervention and using this to strategically improve implementation design.

*Blending active ingredients as a recipe for success-* This study developed an understanding of the mechanisms of impact of the intervention by describing three assumed pathways; (i) awareness generation breaks down stigma to allow for open SRH discussions, (ii) changes in parenting practices facilitates communication, and (iii) communities share responsibilities to facilitate SRH communication. The role authoritarian parenting plays in driving SRH risk [26, 37] and the importance of generating awareness among parents [38, 39] has been expounded in prior literature on parent focused SRH interventions. However, it is also important to note that these pathways worked in tandem, with caregiver’s recognition of the need for SRH discussions, as well as their improved rapport-building skills together promoting parent-child SRH communication. Similarly, the intervention focus on communal learnings motivated communities to share parenting responsibilities while also encouraging co-parenting couples to overcome gender stereotypes and work together as a team. Therefore, it is recommended to consider the relevance of the active ingredients of interventions and how they work together, and utilise this to improve intervention content.

This process evaluation study was limited by data collection challenges due to the ongoing pandemic and contextual challenges in the study setting. Several participants’ ages were not available (instead an age range is provided), and the planned number of interviews with intervention participants was not attained (data from MSC stories was used to supplement this). The intervention reached 287 participants, which was less than the initially planned output of reaching 500 households and is a small proportion of the estimated population of 6,061 residents (approximately 1,520 households) in the study site [18]. One of the strengths of this study is that data analysis was conducted by researchers who were not part of the implementation team, which reduces the potential for bias. Additionally, the evaluation triangulated data from various sources (document data, interviews, focus group discussions and MSC stories) and a range of stakeholders (intervention participants, researchers and program implementers), Finally, this work addresses the dearth of process evaluations on SRH programs by evaluating the intervention’s implementation processes and pathways of success and provides recommendations for further interventions in this area.

## Conclusion

This study evaluated the delivery of a parent focused SRH communication intervention in Uganda, to provide a deeper understanding of how and why the intervention works, by exploring the implementation processes, contextual factors and mechanisms of impact. The findings can be summarised in three key learnings. Firstly, the implementation success was credited to meaningful community engagement throughout the course of intervention planning and delivery, which improved acceptability and uptake in the face of challenges. Secondly, this study provided an overview of how contextual factors acted as barriers and facilitators for the program and recommends further studies conducting contextual analyses to inform implementation design. Finally, this evaluation recognised the interplay between mechanisms of impact and suggested further research consider such combined impacts when designing intervention content. This study hence adds to the global health evidence-base to inform the design and implementation of complex SRH interventions.

### Electronic supplementary material

Below is the link to the electronic supplementary material.

Supplementary Material 1

## Data Availability

The datasets used and/or analysed during the current study are available from the corresponding author on reasonable request.

## Abbreviations

SRH: Sexual and Reproductive Health
HIV: Human Immunodeficiency Virus
MUST: Mbarara University of Science and Technology
UGent: Ghent University
LMICs: Low-and-Middle Income Countries
HICs: High-Income Countries
MRC: Medical Research Council
CAB: Community Advisory Board
MSC: Most Significant Change
IDIs: In Depth Interviews
FGD: Focus Group Discussion
