# Supplementary material for: Process evaluation of a parent-child communication intervention for adolescent sexual and reproductive health in Uganda
Source: BMC Public Health. 2024 Jan 29;24:319. doi: 10.1186/s12889-023-17513-7 (PMC10826092; doi:10.1186/s12889-023-17513-7)
Supplement: Supplementary file 1 — Supplementary Material 1 [file 12889_2023_17513_MOESM1_ESM.docx]

**Appendix**

**Appendix A- Interview guide for Intervention participants (Parents/ Care-givers)**

(after consent process is complete)

Thank you for agreeing to take part in this interview. I appreciate your time. I am interested in hearing your thoughts and experiences about the parent-child communication program conducted in your community. During this interview, we will discuss different aspects of your experience such as any challenges you faced, factors that helped you and outcomes of taking part of this program. We will be using this information to understand what works best for you and other parents in your community. So your feedback will be very important to making this program successful. There are no right or wrong answers, so please feel free to speak your mind. The interview will be more like a conversation, and it will be nice if you give many examples to describe your experiences.

A few details on the interview before we start. It will take approximately an hour. It will be recorded, and your responses will be transcribed to ensure we accurately reflect your words. Your responses will be confidential with all personal identifying details removed so that your identity remains anonymous. And your participation is entirely voluntary, so you can choose not to answer any question. Do you have any questions before we begin?

General Opening Questions

How did you hear about this program?

What motivated you to join? What were your expectations from the program?

Overall, how would you describe your experience with the program? How regularly have you been able to attend the program?

Acceptability- Let’s discuss the acceptability of the program for parents in your community.

How did you and your community feel about the program?

Probes-

- Do you think it meets parents' needs? In what way?
- Do you think it meets the needs of both fathers and mothers? Could you reflect on this?
- How well does it align with the community’s values (eg religion and culture)?
- How have other parents in your community responded to this program?
- What parts of this program are not well suited for parents in your community? Can you describe this?

How could this program be better suited to your community?

Probes-

- What could be done to improve suitability for both parents (mother and father)?
- What could be done to improve suitability in terms of community values?

How do you see this program being implemented in your community in the future?

Probes-

- What components of the program would you like to continue in the future?
- Could you reflect on a community-led initiative to continue this program?
- What resources would be needed for this to happen?

Contextual Factors- I would like to know more about your experience with the program. Let's start with considering factors that have been helpful to you to attend the program.

What factors supported your participation in the program?

Probes-

- Can you reflect on factors that are part of the intervention?
- The content and goals of the intervention?
- How did the timing and duration of the program influence participation?
- Can you reflect on external factors that may have influenced your participation?
- Can you reflect on the ‘new normal’ that has come with Covid and think about how it influenced your participation?
- What motivated you to keep attending?
- What helped you recognise the need for this intervention?

Can you give me examples of these?

What difficulties you may have experienced during the program?

Probes-

- What challenges did you face to attend the sessions?
- Could you reflect on both internal and external factors?
- What challenges did you face while participating in the session?

How does this affect your participation in the program?

How did you handle the situation?

What could have been done to improve your experience?

Pathways- Let’s discuss the changes you have experienced and how these have come about.

What changes have you noticed since participating in the program?

Can you describe how you have applied what you learned through the program? Please give me an example of a situation?

What challenges did you experience in applying what you learned?

Probes

- What changes have you noticed in your knowledge and attitudes about SRH?
- What changes have you noticed in your role as a parent?
- What changes have you noticed in your communication with your child?
- Can you think of any other changes that have occurred since participating?

According to you, what is the reason for this change?

Probes-

- Could you reflect on how the program influenced this change?
- Could you think of any external factors that may have influenced this change?
- Can you describe how this may have happened?

Before we end, could you share your thoughts on how this program could be changed to improve your experience?

Thank you for your time.

**Appendix B- Interview guide for Researchers**

(after consent process is complete)

Thank you for agreeing to take part in this interview. I appreciate your time. I am interested in hearing your thoughts and experiences about the parent-child communication program conducted in your community. During this interview, we will discuss different aspects of your experience such as any challenges you faced, factors that helped you and outcomes of taking part of this program. We will be using this information to understand what works best for you and other parents in your community. So your feedback will be very important to making this program successful. There are no right or wrong answers, so please feel free to speak your mind. The interview will be more like a conversation, and it will be nice if you give many examples to describe your experiences.

A few details on the interview before we start. It will take approximately an hour. It will be recorded, and your responses will be transcribed to ensure we accurately reflect your words. Your responses will be confidential with all personal identifying details removed so that your identity remains anonymous. And your participation is entirely voluntary, so you can choose not to answer any question. Do you have any questions before we begin?

General Opening Questions

Could you describe your overall experience working on this project?

Probes- what have you learned? What is your role on the project?

Acceptability- Let’s discuss the acceptability of the program for parents in your community.

How suitable is this program for parents in the community?

Probes-

- Do you think it meets parents' needs? In what way?
- Do you think it meets the needs of both fathers and mothers?
- How well does it align with the community’s values?
- How have parents in the community responded to this program?
- What parts of this program are not well suited for parents in your community?

How could this program be better suited to your community?

Probes-

- What could be done to improve suitability for both parents (mother and father)?
- What could be done to improve suitability in terms of community values?
- Could you reflect on the MUST team’s collaboration with the community? How do you see this continuing in the future?
- What resources would be needed for this to happen?
- How would the research team support this process?

Contextual Factors- I would like to know more about your experience with the program.

What factors were helpful in developing and delivering the program?

What has helped participants to engage with the program?
What has helped community facilitators to deliver the program?

Probes-

- Lets reflect on Intervention factors such as the objectives of the program, and the content of the intervention. How did these factors facilitate the development and delivery of the project?
- Now lets reflect on the stakeholders involved, such as the parents, the community, the community facilitators who delivered the intervention, and local leaders. Can you describe how these stakeholders supported the delivery of the project?
- Lets consider external factors, such as societal values, the political situation, and any other contextual factors. How did these factors help in delivering the project?
- Can you give me examples of these?

Can you tell me about challenges you experienced during the program?

Probes-

- What challenges did you face as a researcher in developing the intervention?
  Can you think about some sessions that were difficult to deliver? Why was this?
- Can you describe challenges working with the community facilitators?
- What were the difficulties you experienced in engaging with participants?

what were the challenges faced by participants?

Probes-

challenges in attendance, understanding the content, comfort with the content, interacting/ speaking in the session

How does this issue affect engagement with the program? / How does this issue affect the program's outcomes?

Fidelity- Let’s discuss changes that were made to the intervention since rolling out the program

Could you describe some changes that were made to the intervention?

Can you describe times when you had to make adjustments to the intervention while delivering it?

Can you tell me about changes in the timing or duration of the sessions? Why were these changes made?

probes-

Why were these changes made?

Could you talk about your experience?

How did making adjustments improve the situation?

How did your presence as an observer influence the intervention?

Pathways

What changes have you noticed within yourself since facilitating the program?

What changes have you noticed among parents since working on the program?

Probes-

- What changes have you noticed in parent’s knowledge and attitudes about SRH?
- What changes have you noticed in their communication with their child?
- What changes have you noticed in the community at large?
- Can you think of any other changes that have occurred since participating?

       According to you, what is the reason for this change?

Probes-

- Could you reflect on how the program influenced this change?
- Could you think of any external factors that may have influenced this change?
- Can you describe how this may have happened?

Could you share your thoughts on how this program could be further improved?

Thank you for your time

**Appendix C- Most Significant Change (MSC) story Interview guide**

We are part of the research team from Mbarara University of Science and Technology that is working on the research project titled Improving Adolescent Sexual and Reproductive Health through a Participatory Parent-Child Communication Intervention in Uganda (CPAC Project). The project is being implement in Rwebishekye Parish, Rwanyamahembe, Mbarara District. We are here to capture stories of most significant change that may have resulted from the Parent-Child Communication on SRH intervention/training which you participated in. Your story of most significant change will help us to understand and document the importance of SRH parent-child communication to you as an individual and to the rest of the community.

The stories and the information collected from these interviews will be used for a number of purposes including:

- Learning what has been achieved with the intervention
- Understanding the importance of parent child communication to adolescent sexual and reproductive health (ASRH) outcomes
- Acknowledging and documenting the relevance of the intervention

Participants Details

Name of the Storyteller ________________________

Age of the storyteller ________________(years)

Sex _________________(Male/Female)

Age of the youngest child of the Storyteller________________

Village _________________________

Date____________________________

Confidentiality

We may wish to share your story for reporting and publication, or sharing with other people in this community and beyond including policy makers and other researchers. Do you agree – Yes/No

Do you allow us to write down your story (without your real names) and share with others – Yes/No

Would you like your name on the story? – Yes/No

Would you like your photo on the story? – Yes/No

Questions

1. Tell me how you learned about the parent and child communication intervention and how involved were you?
2. Please take a few minutes to think about the changes that have happened since you started participating in the parent and child communication intervention.
   1. Please share the most significant change that has come as a result of your participation in the parent and child communication training sessions on sexual and reproductive health?
   2. Describe the story in relation to
      1. Communicating SRH issues with your child,
      2. What you think about ASRH (attitude),
      3. what you have learned (knowledge),
      4. parenting (techniques and behavior) and
      5. the general changes that have happened to you as a parent (personal life and family) ()(Using visual aids as probes)
3. Why is this change significant for you?
   1. What is the reason that this change occurred? (Using visual aids- topics, stories from other parents, an event in community, the child, the family, you, something else- lets us know?)
   2. What external factors helped you to achieve this change? What challenges did you face?

**Appendix D- Focus Group Discussion guide for Community Facilitators**

(after consent process is complete)

Thank you for agreeing to take part in this interview. I appreciate your time. I am interested in hearing your thoughts and experiences about the parent-child communication program conducted in your community. During this interview, we will discuss different aspects of your experience such as any challenges you faced, factors that helped you and outcomes of taking part of this program. We will be using this information to understand what works best for you and other parents in your community. So your feedback will be very important to making this program successful. There are no right or wrong answers, so please feel free to speak your mind. The interview will be more like a conversation, and it will be nice if you give many examples to describe your experiences.

A few details on the interview before we start. It will take approximately an hour. It will be recorded, and your responses will be transcribed to ensure we accurately reflect your words. Your responses will be confidential with all personal identifying details removed so that your identity remains anonymous. And your participation is entirely voluntary, so you can choose not to answer any question. Do you have any questions before we begin?

General Opening Questions

How did you hear about this program?

What motivated you to join the program as a facilitator? What were your expectations from the program?

Overall, how would you describe your experience with the program?

Acceptability- Let’s discuss the acceptability of the program for parents in your community.

How was the intervention received by participants?

Probes-

- Do you think it meets parents' needs? In what way?
- Do you think it meets the needs of both fathers and mothers? Could you reflect on this?
- How well does it align with the community’s values (eg religion and culture)?
- How have parents in your community responded to this program?
- What parts of this program are not well suited for parents in your community? Can you describe this?

How could this program be better suited to your community?

Probes-

- What could be done to improve suitability for both parents (mother and father)?
- What could be done to improve suitability in terms of community values?

How do you see this program being implemented in your community in the future?

Probes-

- What components of the program would you like to continue in the future?
- Could you reflect on a community-led initiative to continue this program?
- What resources would be needed for this to happen?

Fidelity- Let’s discuss changes that were made to the intervention since rolling out the program

Could you describe some changes that were made to the intervention? Why were these changes made?

Can you describe times when you had to make adjustments to the intervention while delivering it? Could you talk about your experience?

Can you tell me about changes in the timing or duration of the sessions? Why were these changes made?

Contextual Factors- I would like to know more about your experience with delivering the program.

What factors supported your role as a facilitator in the program?

Can you tell me about challenges you experienced during the program?

Probes-

How did you handle the situation?

What could have been done to support you as a facilitator?

When you think about the challenges you experienced, what motivated you to continue delivering the program?

What facilitators and challenges were experienced by the program's participants

Probes-

- Can you tell me about factors that have helped parents to participate in the program
- Can you reflect on factors that are part of the intervention?
- Can you reflect on external factors that may have influenced their participation?
- Can you give me examples of these?

Now let’s reflect on any difficulties encountered by participants.

Can you tell me about challenges experienced by parents participating in the program? Probe- Could you reflect on both internal and external factors?

Pathways- Let's discuss the changes participants experienced and how these occurred.

What changes have you noticed within yourself since facilitating the program?

What changes have you noticed among parents since facilitating the program?

Probes-

- What changes have you noticed in parent’s knowledge and attitudes about SRH?
- What changes have you noticed in their communication with their child?
- What changes have you noticed in the community at large?
- Can you think of any other changes that have occurred since participating?

According to you, what is the reason for this change?

Probes-

- Could you reflect on how the program influenced this change?
- Could you think of any external factors that may have influenced this change?
- Can you describe how this may have happened?

Before we end, could you share your thoughts on how this program could be changed to improve your experience?

Thank you for your time.

**Appendix E- Qualitative Interviews Themes and Codes**

| **Evaluation areas** | **Themes** | **Codes** |
| --- | --- | --- |
| Implementation | Community engagement | Participatory content development |
|  |  | Community involved in planning |
|  |  | Community-centric delivery and mobilisation |
|  | Rapport with delivery agents | Relationship with facilitators |
|  |  | Cultural proximity of community facilitators |
|  |  | Relationship with research team |
|  | Personal factors | Conducive living/ occupational circumstances |
|  |  | Personal motivation |
|  | Implementation barriers | Disruptions to planned delivery |
|  |  | Infrastructure challenges |
| Contextual Factors | Parenting norms | Authoritarian parenting |
|  |  | Gendered parenting roles |
|  | Perceptions and Attitudes to Youth SRH | Communication gap around SRH |
|  |  | Parents unaware of adolescent’s needs or risk |
|  |  | Stigma around SRH and SRH discussions |
|  |  | Risk-focus in SRH discussions |
|  | Poverty | Lack of time for parenting |
|  |  | Lack of necessary resources |
|  | Covid19 | |
|  | Prior research in the communities | |
| Mechanisms of Impact | Overcoming stigma through awareness | Appreciation for SRH information content |
|  |  | Recognition of adolescents SRH needs |
|  |  | Overcoming SRH stigma |
|  | Rethinking parenting styles | Parenting styles content |
|  |  | Appreciation for communication skills training |
|  |  | Reframing of parent-child relationships |
|  |  | Changing communication styles |
|  | Shared learnings and responsibilities | Group learning and discussion format |
|  |  | Sharing experiences and learnings |
|  |  | Recognising community role in parenting |
|  | Shifting gendered roles | Content on male parenting responsibilities |
|  |  | Changing communication between parents |
|  |  | Changing interpersonal relationships |
